# Curcumin Supplementation and Vascular and Cognitive Function in Chronic Kidney Disease: A Randomized Controlled Trial

**DOI:** 10.3390/antiox13080983

**Published:** 2024-08-14

**Authors:** Colin J. Gimblet, Nicholas T. Kruse, Katharine Geasland, Jeni Michelson, Mingyao Sun, Patrick Ten Eyck, Cari Linkenmeyer, Safur Rehman Mandukhail, Matthew J. Rossman, Meenakshi Sambharia, Michel Chonchol, Manjula Kurella Tamura, Douglas Seals, Karin F. Hoth, Diana Jalal

**Affiliations:** 1 Division of Nephrology, Department of Internal Medicine, Carver College of Medicine, University of Iowa, 375 Newton Rd, Iowa City, IA 52246, USA; mingyao-sun@uiowa.edu (M.S.); safur-rehman@uiowa.edu (S.R.M.); meenakshi-sambharia@uiowa.edu (M.S.); diana-jalal@uiowa.edu (D.J.); 2 College of Health Professionals, Central Michigan University, Mount Pleasant, MI 48859, USA; 3 Institute for Clinical and Translational Science, University of Iowa, Iowa City, IA 52246, USA; katharine-geasland@uiowa.edu (K.G.); jeni-michelson@uiowa.edu (J.M.); patrick-teneyck@uiowa.edu (P.T.E.); 4 Department of Psychiatry, University of Iowa, Iowa City, IA 52246, USA; carinda-linkenmeyer@uiowa.edu (C.L.); karin-hoth@uiowa.edu (K.F.H.); 5 Department of Integrative Physiology, University of Colorado Boulder, Boulder, CO 80309, USA; matthew.rossman@colorado.edu (M.J.R.); douglas.seals@colorado.edu (D.S.); 6 Division of Renal Diseases and Hypertension, University of Colorado Anschutz Medical Campus, Aurora, CO 80045, USA; michel.chonchol@cuanschutz.edu; 7 Division of Nephrology, Department of Medicine, Stanford University School of Medicine, Palo Alto, CA 94305, USA; mktamura@stanford.edu; 8 Geriatric Research, Education and Clinical Center, Veterans Affairs Palo Alto, Palo Alto, CA 94304, USA; 9 Iowa Neuroscience Institute, University of Iowa, Iowa City, IA 52246, USA; 10 Iowa City VA HCS, Iowa City, IA 52246, USA

**Keywords:** CKD, curcumin, vascular function, oxidative stress

## Abstract

Chronic kidney disease (CKD) increases the risk of cardiovascular disease and cognitive impairment. Curcumin is a polyphenol that improves vascular and cognitive function in older adults; however, its effects on vascular and cognitive function in patients with CKD are unknown. We hypothesized that curcumin supplementation would improve vascular and cognitive function in patients with CKD. Eighty-eight adults diagnosed with stage 3b or 4 CKD (aged 66 ± 8 years, 75% male) participated in a 12-month, randomized, double-blind, placebo-controlled study to test the effects of curcumin (Longvida^®^, 2000 mg/day) on vascular and cognitive function. Our primary outcome was brachial artery flow-mediated dilation (FMD). Our secondary outcomes were nitroglycerin-mediated dilation, carotid–femoral pulse wave velocity (cfPWV), and cognitive function assessed via the NIH Toolbox Cognition Battery. At baseline, the mean estimated glomerular filtration rate was 34.7 ± 10.8, and the median albumin/creatinine ratio was 81.9 (9.7, 417.3). A total of 44% of participants had diabetes. Compared with placebo, 12 months of curcumin did not improve FMD (median change from baseline was −0.7 (−2.1, 1.1) and −0.1 (−1.5, 1.5) for curcumin and placebo, respectively, with *p* = 0.69). Similarly, there were no changes in nitroglycerin-mediated dilation, cfPWV, or cognitive outcomes. These results do not support chronic curcumin supplementation to improve vascular and cognitive function in patients with CKD.

## 1. Introduction

Chronic kidney disease (CKD) represents a public health epidemic, affecting more than 10 percent of the global population [1,2]. Patients with CKD are more likely to die from cardiovascular disease (CVD) than to progress to end-stage kidney disease [3]. Moreover, patients with CKD have a greater risk of developing CVD and cognitive impairment than the general population [4,5,6]. Therefore, interventions that reduce CVD risk and improve cognitive function in patients with CKD are desperately needed.

Vascular dysfunction is an important non-traditional risk factor purported to play a critical role in the pathogenesis of both CVD and cognitive impairment in individuals with CKD [7,8]. Specifically, dysfunction of the vascular endothelium and stiffening of the aorta are both present in CKD [9,10,11] and predict higher CVD risk in this population [10,12]. Additionally, cerebral small vessel disease and aortic stiffness contribute to lower cognition in patients with CKD [13,14,15,16]. Thus, it is plausible that improving vascular function would not only reduce the risk of CVD, but also that it would augment cognitive function in patients with CKD.

Polyphenols are naturally occurring compounds with potent antioxidant and anti-inflammatory properties and protect against the development of several chronic diseases including CVD [17,18]. Importantly, oxidative stress and inflammation promote vascular dysfunction in patients with CKD [19]. Curcumin is a polyphenol extracted from the Indian spice turmeric that has been shown to lower circulating biomarkers of oxidative stress and inflammation in humans [20,21]. Curcumin supplementation was found to improve endothelial function and reduce aortic stiffness in aged mice and to improve endothelial function in older adults, in part, by suppressing oxidative stress [22,23]. In addition, several studies indicate that curcumin supplementation may improve selective domains of cognitive function in older adults [24,25,26]. However, no study to date has evaluated if curcumin supplementation improves vascular and cognitive function in patients with CKD. Here, we hypothesized that 12-month curcumin supplementation would improve endothelial and cognitive function and reduce aortic stiffness in patients with CKD.

## 2. Methods

### 2.1. Study Population

Study participants were recruited between June 2018 and February 2022 from the Renal Clinics at the University of Iowa Hospital and Clinics (UIHC) and the Iowa City Veterans Affairs Health Care System (VA HCS). Inclusion criteria consisted of individuals between ages 45 and 74 years, able to provide informed consent, and with an estimated glomerular filtration rate (eGFR) of 20–44 mL/min/1.73 m^2^ and a body mass index (BMI) of less than 35 kg/m^2^. Exclusion criteria included consuming a diet rich in curcumin or taking curcumin supplements in the past 12 months, life expectancy of less than one year, current or anticipated pregnancy, breastfeeding, uncontrolled hypertension, systolic heart failure, severe liver disease, acute infection, antibiotic therapy, hospitalization within the last 3 months, immunosuppressive therapy within the past year, or current participation in another clinical trial. The University of Iowa and the Iowa City VA HCS Institutional Review Boards approved this study, which was conducted per the Declaration of Helsinki. All participants were given documentation of the informed consent process and signed before study participation. (NCT03223883).

### 2.2. Study Procedures

This study followed a randomized, double-blind, placebo-controlled, parallel-arm design. Participants were randomized 1:1 to curcumin or placebo and stratified based on age ≥ 55 years and baseline diabetes status. The study drug, solid lipid curcumin particle capsules (Longvida^®^), and a matching placebo were provided by Verdure Sciences. Subjects were enrolled via a blinded coordinator. The randomization table was generated via Excel by the study biostatistician. For the research team to remain blinded, the study subject group allocation, study drug packaging, labeling, and dispensation were managed by the Investigational Drug Service Pharmacy at UIHC. Upon randomization, participants were instructed to consume Longvida^®^ (2000 mg/day) or placebo once daily for 12 months. All study measurements and procedures were performed in the Clinical Research Unit at UIHC. Vascular outcomes were evaluated at baseline, 6 months, and 12 months whereas cognitive function and blood biomarker outcomes were evaluated at baseline and 12 months.

Adherence was determined at the end of 12 months by pill count. The number of pills ingested was calculated as the number of pills provided/the number of pills returned. Percent adherence was then calculated as number of pills ingested/number of pills provided × 100.

### 2.3. Clinical Characteristics

Race, ethnicity, smoking status, and preexisting CVD were evaluated by questionnaire. Smoking status was defined as a history of smoking (current or former) or no history of smoking. Preexisting CVD was defined as a history of myocardial infarction, stroke, arrhythmia, or heart failure. Blood pressure was measured in the seated position and obtained in triplicate, following 10 min of quiet rest. BMI was calculated by dividing weight by height in kg/m^2^. Clinical labs were measured at the UIHC diagnostics laboratories and included fasting lipid panel, hemoglobin A_1c_ (HbA_1c_), creatinine, cystatin C, and urinary albumin/creatinine ratio (UACR). eGFR was calculated from cystatin C using the CKD-EPI formula as previously described [27].

### 2.4. Primary Outcome

The primary outcome of our study was the change in brachial artery flow-mediated dilation (FMD) from baseline to 12 months. FMD was measured by high-resolution ultrasonography (GE Logiq E9) as previously described by our lab [28,29]. Briefly, following the baseline diameter measurements of the brachial artery, a cuff placed on the upper forearm was inflated to 250 mm Hg for 5 min. After 5 min, the cuff was rapidly deflated, and measurements were continuously recorded for an additional 2 min. Baseline and deflation images were analyzed using offline software (Vascular Analysis Tools 5.5, Medical Imaging Applications, LLC, Coralville, IA, USA). FMD was calculated as percent change in brachial artery diameter using the following equation:

(1) $$FMD=\frac{∆\;diameter\;}{baseline\;diameter}\times 100$$

### 2.5. Secondary Outcomes

Peak nitroglycerin-mediated dilation was determined by imaging brachial artery vasodilation for 10 min after administration of 0.4 mg sublingual nitroglycerin (GE Logiq E9). Peak nitroglycerin dilation was calculated using the same equation used to assess FMD.

Aortic stiffness was determined via carotid–femoral pulse wave velocity (cfPWV; NIHem workstation; Cardiovascular Engineering, Inc., Norwood, MA, USA) [30]. Briefly, an applanation tonometer was used to collect the carotid and femoral artery waveforms from their respective pulse sites. Upon analysis, carotid and femoral artery waveforms were gated to the R-wave of the ECG to determine the foot-to-foot time delay. Carotid–femoral transit distance was computed as the distance between the suprasternal notch and the femoral waveform pulse site minus the distance from the suprasternal notch to the carotid waveform pulse site. cfPWV was calculated as the carotid–femoral transit distance divided by the carotid–femoral foot-to-foot time delay.

The NIH Toolbox Cognition Battery, a validated multidimensional assessment of cognition, was utilized to evaluate 4 cognitive domains: (1) processing speed (Pattern Comparison Processing Speed, Oral Symbol Digit), (2) executive function (Flanker Inhibitory Control and Attention, Dimensional Card Sort), (3) memory (Picture Sequence Memory, Rey Auditory Verbal Learning Test Immediate Memory), and (4) language (Picture Vocabulary, Oral Reading Recognition) [31]. The NIH Toolbox Cognition Battery Version 2 was administered to our participants by a single trained research assistant (blinded to subject randomization) in a quiet, private exam room for approximately 45 min. Raw scores were age-adjusted and expressed as standard scores (mean of 100 and standard deviation of 15) by the NIH Toolbox scoring software (https://nihtoolbox.org/). Domain scores were calculated as the mean of the normatively derived standard scores for all tests in that domain. Thus, a domain score of 100 indicates exactly average performance whereas a score of 85 indicates performance that is 1 SD below the mean relative to healthy age-matched peers.

Venous blood samples were obtained at baseline and 12 months for the determinations of plasma interleukin-6 (IL-6) and oxidized low-density lipoprotein (oxLDL) as biomarkers of inflammation and oxidative stress, respectively. IL-6 (R&D Systems, Catalog #D6050B) and oxLDL (ALPCO Diagnostics, Catalog #30-7810) were quantified using commercially available enzyme-linked immunosorbent assays performed according to kit instructions.

### 2.6. Statistical Analysis and Power Calculation

Descriptive statistics were calculated by study group randomization for demographics, baseline measures, and outcome variables. Normally distributed data are presented as mean ± SD, non-normally distributed data as median (interquartile range), and categorical data as count (percentage of participants). Variables that were normally distributed were compared via the two-sample *t*-test whereas variables that were not normally distributed were compared via the Wilcoxon Rank-Sum test. Categorical measures were assessed using Fisher’s exact test. A two-sample *t*-test or a Wilcoxon Rank-Sum test was applied to evaluate if curcumin improved the primary and secondary outcomes. All outcome analyses were based on the “intent-to-treat” (ITT) principle.

The power calculation was based on the primary outcome (change in FMD after 12 months) via a two-sample *t*-test of change using PASS [32]. Our preliminary data in older adults without CKD (22) yielded a mean and standard deviation (SD) change in FMD of 1.25 (0.88) with curcumin and 0.08 (0.40) with placebo. We believed that the change in CKD would be smaller and assumed a mean (SD) of 0.7 (0.9) for the change in FMD with curcumin. We assumed 0.10 (0.4) as the mean (SD) change in FMD with placebo [33]. With a target power of 95% and 2-sided alpha 0.05, we calculated a sample size of 37 participants per group is required. With an assumed attrition rate of 18%, we sought to recruit 44 subjects per group to achieve the study objective.

## 3. Results

### 3.1. Clinical Characteristics

**Figure 1:**
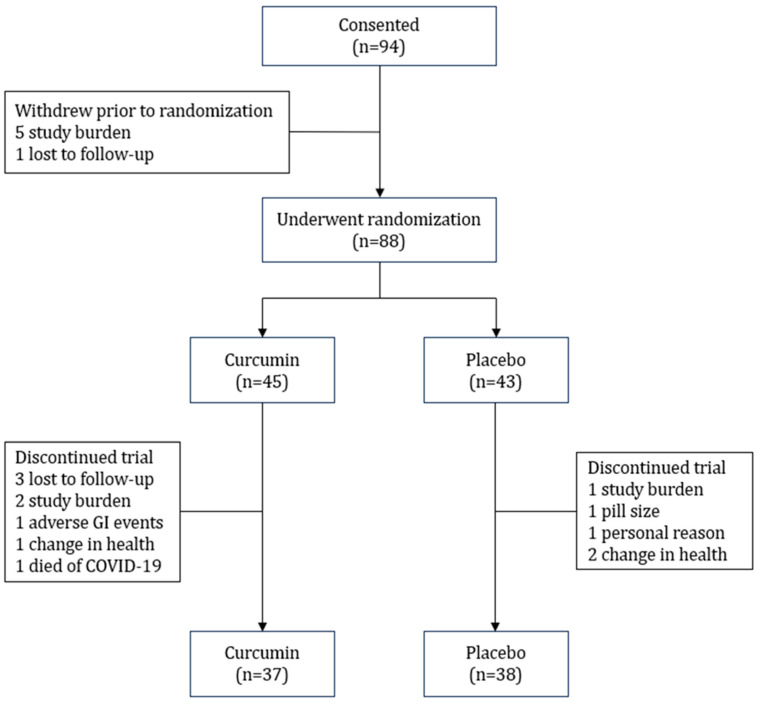
Study schema.

A total of 94 participants with stage 3b or 4 CKD were screened and consented; of those, 6 withdrew before randomization and the remaining 88 were randomized. Of the 45 participants randomized to receive curcumin, 8 (18%) withdrew from this study. Of the 43 participants randomized to receive placebo, 5 (12%) withdrew from this study (Figure 1). Baseline clinical characteristics are presented in Table 1. On average, the participants were 66 ± 8 and 65 ± 8 years of age in the curcumin and placebo groups, respectively. The curcumin group was 78% male, while the placebo group was 72% male. No differences were noted in kidney function or albuminuria between the groups. Most participants were overweight or obese according to BMI. However, we observed no differences between groups in BMI or glycemic control. Diabetes was present in 44% and 45% of the participants in the curcumin and placebo groups, respectively. In both groups, the majority of the participants had no prior history of CVD. Lastly, there were no between-group differences in cognitive function at baseline (Table 2).

### 3.2. Primary Outcome

**Figure 2:**
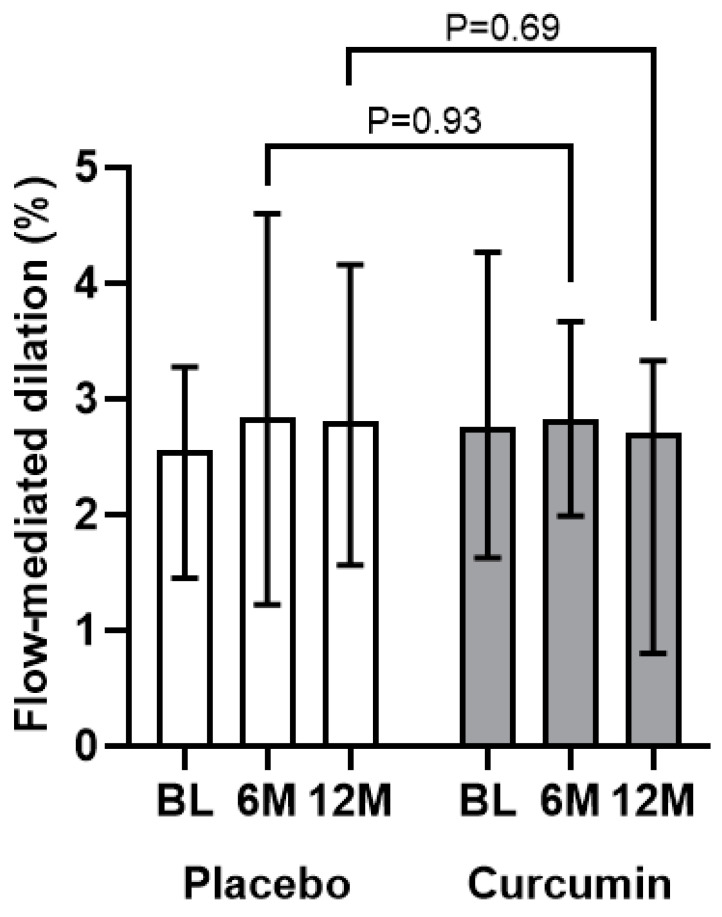
Brachial artery flow-mediated dilation at baseline, 6 months, and 12 months according to study group. Data are presented as median (interquartile range). A Wilcoxon Rank-Sum test was used to compare the change in brachial artery flow-mediated dilation between groups at 6 and 12 months. BL, baseline; 6M, 6 months; 12M, 12 months.

As shown in Figure 2 and Supplemental Table S1, curcumin supplementation at 2000 mg per day for 12 months did not result in an increase in brachial artery FMD compared with placebo (−0.7 [−2.1, 1.1] versus −0.1 [−1.5, 1.5]; *p* = 0.69). When evaluating the interaction term for the change in brachial artery FMD between groups over time, we observed no interaction with age (*p* = 0.66), diabetes status (*p* = 0.16), or eGFR (*p* = 0.09). Similarly, no significant effect of curcumin was noted on brachial artery FMD at the 6-month time-point (Figure 2). (The 6-month change in FMD was 0.02 [−1.6, 1.1] in the curcumin group and 0.74 [−1.5, 1.7] in the placebo group with the *p* value for the between-group comparison = 0.93.)

### 3.3. Secondary Outcomes

The secondary outcomes at the month 12 timepoint are shown in Table 3 and Supplemental Table S1. There was no difference in nitroglycerin-mediated dilation with curcumin compared with the placebo group at 12 months. Additionally, no between-group differences were observed for the change in cfPWV (aortic stiffness) after supplementation at 12 months. Of note, there was no significant improvement in nitroglycerin-mediated dilation and cfPWV at 6 months (Supplemental Table S1). When compared with the placebo group, curcumin did not improve processing speed, executive function, memory, or language at 12 months (Table 3). Moreover, 12-month curcumin supplementation did not reduce oxLDL when compared with placebo (Table 3). However, between-group comparisons revealed that 12-month curcumin supplementation reduced IL-6 more than placebo (Table 3).

Systolic blood pressure, diastolic blood pressure, HbA1c, and UACR were unchanged in both the curcumin and placebo groups (Table 4 and Supplemental Table S1). eGFR increased slightly in the curcumin group by 0.75 ± 5.7 and decreased in the placebo group by 1.30 ± 5.2, but the between-group difference was not significant (*p* = 0.22).

### 3.4. Adverse Events and Adherence

There was no difference in reported adverse events between the curcumin and placebo groups (Table 5). Importantly, no serious adverse events were reported with curcumin supplementation. One subject in the curcumin group died due to COVID-19. There were no differences in nausea, vomiting, or dizziness between the curcumin and placebo groups. Six (13.3%) participants reported abdominal pain in the curcumin group compared with two (4.7%) in the placebo group, but this did not achieve statistical significance (*p* = 0.27). Median study supplement adherence was high in both groups [99% (96, 100) for curcumin and 98% (95, 100) for placebo (*p* = 0.28)].

## 4. Discussion

To our knowledge, this is the first study to assess the effects of 12-month curcumin supplementation (Longvida, 2000 mg/day) on vascular and cognitive function in patients with stage 3b or 4 CKD. While we found that 12-month supplementation with curcumin is safe and well-tolerated among the participants, we observed no between-group difference in our primary outcome of brachial artery FMD. Additionally, we noted no significant between-group differences in our secondary outcomes of nitroglycerin-medicated dilation, aortic stiffness, cognitive function, or plasma oxLDL after supplementation. The 12-month curcumin supplementation did, however, reduce plasma IL-6 more than placebo. Our findings indicate that curcumin does not improve vascular or cognitive function in patients with stage 3b or 4 CKD. The anti-inflammatory properties of curcumin in patients with CKD may warrant further investigation.

Evidence supports an emerging role of curcumin supplementation in improving vascular health in aging and disease [34,35,36,37]; however, these findings are not uniform [36,38,39]. Supplementation with curcumin has been shown to lower oxidative stress [40], restore endothelial function, and reduce aortic stiffness in animal models of healthy aging [23], in addition to decreasing oxidative stress in a rat model (5/6 nephrectomy) of CKD [41]. In healthy older adults, 12-week supplementation with curcumin (Longvida, 2000 mg/day) attenuated oxidative stress and improved endothelial function, although it did not improve aortic stiffness [22]. However, recent translational evidence in children and young adults with autosomal dominant polycystic kidney disease (ADPKD) and normal kidney function reported no significant benefit on endothelial function or large artery stiffness after 12 months of curcumin supplementation [38]. Consistently, we observed that curcumin did not increase brachial artery FMD or reduce aortic stiffness in our participants with moderate–advanced CKD. Notably, our study population had a markedly higher cardiovascular risk profile compared with the older adults in the 12-week curcumin study [22], which may explain our disparate findings regarding the effect of curcumin on endothelial function. Collectively, our findings in conjunction with the recent evidence in patients with ADPKD suggest that long-term use of curcumin does not elicit additional benefits on vascular function in those with CKD.

In the present investigation, we evaluated cognitive function using the NIH Toolbox Cognition Battery, including measures of processing speed, executive function, memory, and language [42]. Several animal models have demonstrated that curcumin attenuates disease-associated cognitive impairments [43,44,45], and improves cognitive function, including learning and memory, in rodent models of accelerated aging, as well as in healthy older rodents [46,47]. However, the effects of curcumin on cognitive function in middle-aged and older adults have been inconsistent. In healthy older adults, 4-week curcumin therapy increased the number of correct responses in a serial subtraction task after adjusting for participant demographics [26]. Additionally, 12-week curcumin supplementation improved performance on an oral reading recognition task in healthy older adults [24]. Moreover, 18-month curcumin supplementation enhanced memory and attention in non-demented older adults [25]. Conversely, 12-month curcumin therapy did not improve measures of cognition in community-dwelling older adults [48]. Our investigation found no changes in cognitive function across any domain using the NIH Toolbox Cognition Battery. These findings, consistent with our observation that curcumin did not enhance vascular function in our study population, suggest that the beneficial effects of curcumin seen in animal models of aging and older adults may not extend to individuals with kidney disease.

The current study found no effect of chronic curcumin supplementation on kidney function or albuminuria, although there was a small, but insignificant, improvement in eGFR after curcumin compared with placebo, despite no observed change in endothelial function. Considering that inflammation and oxidative stress contribute to the development and progression of CKD [49], curcumin may slow CKD progression through mechanisms independent of changes in endothelial function such as reduced oxidative stress and inflammation within the kidney. Nuclear factor erythroid 2-related factor 2 (Nrf2) is a ubiquitous transcription factor that responds to reactive oxidative species overproduction and modulates oxidative stress [50]. In male Sprague Dawley rats with adenine-induced CKD, curcumin lowered systemic biomarkers of inflammation including IL-6, and induced Nrf2 in the kidney tissue in conjunction with reduced tubular necrosis and interstitial fibrosis [51]. In another study, curcumin attenuated the activation of the pro-inflammatory transcription factor nuclear factor-κB (NF-κB) in the kidneys [52]. Such studies suggest that curcumin may confer nephroprotection. However, our study was not designed to evaluate kidney disease progression as an outcome.

Strengths of this investigation include the double-blind randomized placebo-controlled design. Furthermore, our investigation was adequately powered to detect a clinically meaningful difference in our primary outcome (brachial artery FMD). Lastly, this study was completed with no serious adverse events and with a smaller than anticipated number of withdrawals.

Our investigation, however, is not without limitations. First, we did not investigate earlier (stages 1–2) or later stages of kidney disease (e.g., end-stage kidney disease). Nevertheless, our findings regarding the effect of long-term curcumin supplementation on indices of vascular and cognitive function, in conjunction with the recent evidence in young individuals with ADPKD and mild CKD [38], suggest that curcumin supplementation does not elicit a robust response in these measures in patients with CKD. It is unlikely that individuals with greater impairments in kidney function (i.e., stage 5/end-stage kidney disease) would benefit from curcumin whereas those with stage 3b and 4 did not. Generally, the “uremic switch” could confound the influence of nutraceutical therapeutic strategies aimed at targeting the endothelium and improving vascular function in advanced CKD [34,53]. Second, cognitive impairment was not an inclusion criterion. As a result, our study participants exhibited no evidence of cognitive impairment at baseline. Whether curcumin supplementation might have exerted benefits in individuals with clear evidence of cognitive impairment is unclear. Lastly, we did not measure circulating plasma curcumin metabolites in our subject population. However, in this study, we used a third-generation curcumin formulation (Longvida^®^) [54], a solid lipid curcumin particle preparation with demonstrated enhanced bioavailability of unconjugated curcumin compared with standard curcuminoid extracts [55].

## 5. Conclusions

Collectively, our findings demonstrate that 12 months of curcumin supplementation does not improve vascular or cognitive function in patients with stage 3b or 4 CKD. These data indicate that adding curcumin supplements to a typical Western diet will likely not enhance vascular or cognitive performance in middle-aged or older adults with CKD. Further study may be warranted to explore the potential effects of curcumin on inflammation and kidney disease progression in individuals with CKD.

## Figures and Tables

**Table 1:** Baseline characteristics of adults with stage 3b or 4 CKD according to study group.

Variable	Curcumin (*n* = 45)	Placebo (*n* = 43)	*p* Value
Age, years	66 ± 8	65 ± 8	0.17
Sex, no. (%)			0.63
Female	10 (22)	12 (28)	
Male	35 (78)	31 (72)	
Race, no. (%)			0.9
White	41 (91)	40 (93)	
Black	4 (9)	1 (2)	
Multi-racial	0 (0)	1 (2)	
Unknown	0 (0)	1 (2)	
Ethnicity, no. (%)			0.49
Hispanic or Latino	0 (0)	1 (2)	
Non-Hispanic or Latino	45 (100)	42 (98)	
History of smoking, no. (%)			0.52
Yes	18 (44)	22 (51)	
No	23 (56)	21 (49)	
History of CVD, no. (%)			
Arrhythmia	3 (7)	5 (12)	0.48
Myocardial infarction	5 (11)	3 (7)	0.71
Stroke	2 (4)	4 (9)	0.42
Diabetes mellitus, no. (%)			0.93
Type I	1 (2)	2 (5)	
Type II	19 (42)	17 (40)	
Anti-HTN medication, no. (%)			
ACE inhibitor/ARB	26 (42)	14 (33)	0.39
Calcium channel blockers	15 (33)	15 (35)	0.99
Beta blockers	7 (16)	7 (16)	0.99
Diuretics	7 (16)	7 (16)	0.99
Diabetic medication, no. (%)			
SGLT2 inhibitor	3 (7)	2 (5)	0.99
BMI, kg/m^2^	30.0 ± 4.0	29.5 ± 4.5	0.86
Systolic BP, mm Hg	137 ± 21	135 ± 24	0.44
Diastolic BP, mm Hg	74 ± 14	70 ± 12	0.23
HbA_1c_, %	7.3 ± 1.2	7.5 ± 1.4	0.71
Total cholesterol, mg/dL	165 ± 43	153 ± 41	0.20
Triglycerides, mg/dL	129 (88, 181)	137 (93, 182)	0.80
LDL, mg/dL	91 ± 39	78 ± 33	0.32
HDL, mg/dL	44.5 ± 12.1	43.6 ± 13.0	0.52
Creatinine, mg/dL	2.1 ± 0.4	1.9 ± 0.5	0.13
Cystatin C, mg/dL	1.9 ± 0.4	1.9 ± 0.5	0.51
eGFR, ml/min/1.73 m^2^	33 ± 8	35 ± 8	0.23
UACR, mg/g	66 (12, 368)	84 (9, 443)	0.30
IL-6, pg/mL	7.4 (4.4, 11.1)	6.9 (3.7, 9.8)	0.5
oxLDL, ng/mL	28.4 (7.0, 54.8)	45.3 (26.1, 157.2)	0.08
Brachial artery FMD, %Δ	2.8 (1.7, 4.2)	2.6 (1.5, 3.3)	0.73
Nitroglycerin dilation, %Δ	14.7 ± 6.9	13.2 ± 6.1	0.34
cfPWV, m/s	10.1 ± 2.3	10.7 ± 2.9	0.34

Variables are presented as mean ± standard deviation, median (interquartile range), or number (percentage). Variables that were normally distributed were compared via the two-sample *t*-test whereas variables that were not normally distributed were compared via the Wilcoxon Rank-Sum test. Categorical measures were assessed using Fisher’s exact test. CVD, cardiovascular disease; SGLT2, Sodium-Glucose Transport Protein-2; BMI, body mass index; BP, blood pressure; HbA_1c_, hemoglobin A_1c_; LDL, low-density lipoprotein; HDL, high-density lipoprotein; eGFR, estimated glomerular filtration rate; UACR, albumin/creatinine ratio, IL-6, interleukin-6; oxLDL, oxidized low-density lipoprotein; FMD, flow-mediated dilation; cfPWV, carotid–femoral pulse wave velocity.

**Table 2:** Baseline cognitive function of adults with stage 3b or 4 CKD according to study group.

Variable	Curcumin (*n* = 45)	Placebo (*n* = 43)	*p* Value
Processing speed	100.4 ± 10.4	97.4 ± 10.9	0.19
Executive function	97.6 ± 10.4	96.1 ± 9.9	0.50
Memory	97.7 ± 10.9	99.7 ± 9.9	0.38
Language	98.9 ± 10.8	101.5 ± 11.3	0.28

Cognitive function domains were calculated as age-adjusted standard scores with a mean of 100 and a standard deviation of 15. Variables are presented as mean ± standard deviation and compared using the two-sample *t*-test.

**Table 3:** Change in secondary outcomes according to group.

Variable	Curcumin	Placebo	Between-Group *p* Value
Nitroglycerin-mediated dilation, %	−1.33 ± 7.73	−1.25 ± 6.5	0.97
cfPWV, m/s	0.28 ± 2.4	0.36 ± 4.2	0.94
Processing speed	−0.87 ± 8.9	1.78 ± 7.0	0.16
Executive function	2.62 ± 9.0	2.25 ± 9.5	0.86
Memory	2.19 ± 9.2	2.62 ± 8.5	0.83
Language	−0.53 ± 5.8	−1.33 ± 4.5	0.51
IL-6, pg/mL	−0.56 (−4.1, 0.81)	1.2 (−1.1, 5.0)	0.04 *
oxLDL, ng/mL	−6.23 (−17.4, 6.69)	−21.5 (−51.0, 8.06)	0.20

Variables are presented as mean ± standard deviation or median (interquartile range). A two-sample *t*-test (normally distributed variables) and a Wilcoxon Rank-Sum test (non-normally distributed variables) were used to compare the change in outcome variables groups over time. cfPWV, carotid–femoral pulse wave velocity; IL-6, interleukin-6; oxLDL, oxidized low-density lipoprotein. * *p* < 0.05.

**Table 4:** Change in cardiovascular risk factors according to group.

Variable	Curcumin	Placebo	Between-Group *p* Value
Systolic BP, mm Hg	−2.62 ± 14.7	−3.30 ± 19.8	0.48
Diastolic BP, mm Hg	−3.76 ± 15.8	−1.58 ± 7.9	0.69
HbA1c, %	−0.03 ± 0.84	0.09 ± 1.25	0.27
eGFR, ml/min/1.73 m^2^	0.75 ± 5.7	−1.30 ± 5.2	0.22
UACR, mg/g	0.00 (−5.5, 35.1)	1.15 (−11.0, 17.4)	0.78

Variables are presented as mean ± standard deviation or median (interquartile range). A two-sample *t*-test (normally distributed variables) and a Wilcoxon Rank-Sum test (non-normally distributed variables) were used to compare the change in outcome variables groups over time. BP, blood pressure; eGFR, estimated glomerular filtration rate; UACR, albumin/creatinine ratio.

**Table 5:** Adverse events according to group.

Variable	Curcumin (*n* = 45)	Placebo (*n* = 43)	*p* Value
Any adverse event	27 (60.0)	21 (48.8)	0.39
Nausea	7 (15.6)	6 (14.0)	0.99
Vomiting	4 (8.9)	6 (14.0)	0.52
Abdominal pain	6 (13.3)	2 (4.7)	0.27
Dizziness	10 (22.2)	8 (18.6)	0.79

Data are presented as number (percentage of participants). A Fisher’s exact test was used to compare adverse events between groups.

## Data Availability

If accepted for publication, the raw de-identified data including clinical, vascular, and cognitive domain variables will be publicly available via figshare at http://figshare.com.

## Supplementary Materials

The following supporting information can be downloaded at: https://www.mdpi.com/article/10.3390/antiox13080983/s1. Table S1: Outcome and clinical variables by group at each time point.
