# Supplementary material for: Curcumin Supplementation and Vascular and Cognitive Function in Chronic Kidney Disease: A Randomized Controlled Trial
Source: Antioxidants (Basel). 2024 Aug 14;13(8):983. doi: 10.3390/antiox13080983 (PMC11352164; doi:10.3390/antiox13080983)
Supplement: Supplementary file 1 [file antioxidants-13-00983-s001.zip › antioxidants-3121599-supplementary.pdf]

Supplemental Table S1. Outcome and clinical variables by group at each time point

	Curcumin (n=45)			Placebo (n=43)		
Variable	Baseline	6-months	12-months	Baseline	6-months	12-months
FMD, %Δ	2.8 (1.7,4.2)	2.8 (2.1,3.6)	2.7 (0.8,3.3)	2.6 (1.5,3.3)	2.8 (1.3,4.6)	2.8 (1.6,4.1)
Nitroglycerin-mediated dilation, %Δ	14.7±6.9	13.1±7.5	14.7±6.0	13.2±6.1	16.1±9.3	11.0±7.1
cfPWV, m/s	10.1±2.6	9.9±2.8	10.3±2.7	10.7±2.9	10.4±3.4	11.3±4.5
Processing speed	100.4±10.4	-	99.4±12.9	97.4±10.9	-	99.4±10.8
Executive function	97.6±10.4	-	99.7±10.4	96.1±9.9	-	99.1±10.7
Memory	97.7±10.9	-	100.4±10.3	99.7±9.9	-	102.3±12.3
Language	98.9±10.8	-	98.2±10.8	101.5±11.3	-	100.1±12.1
IL-6, pg/mL	7.4 (4.4, 11.1)	8.2 (4.8,10.4)	6.3 (4.0,9.0)	6.9 (3.7, 9.8)	7.4 (5.5,13.0)	7.9 (4.5,13.4)
oxLDL, ng/mL	28.4 (7.0, 54.8)	20.8 (2.9,57.4)	14.8 (4.6, 52.7)	45.3 (26.1, 157)	42.4 (13.7,106.3)	36.7 (16.6,114)
Systolic BP, mm Hg	137±21	140±17	134±18	135±24	133±23	132±22
Diastolic BP, mm Hg	74±14	74±9	70±12	72±11	71±10	69±11
HbA1c, %	7.3±1.2	6.9±0.8	7.2±0.9	7.5±1.4	7.6±1.2	7.5±0.9
eGFR, ml/min/1.73m^2	33.6±8.8	33.1±9.2	34.6±10.9	36.0±12.6	35.5±11.1	36.5±12.7
UACR, mg/g	65.9 (11.5,368)	62.3 (11,288)	67.3 (9.1,315)	83.8 (8.8,443)	35.6 (6.5,476)	36.3 (6.9,372)

Variables are presented as mean ± standard deviation or median (interquartile range). Cognitive function domains were calculated as age-adjusted standard scores with a mean of 100 and a standard deviation of 15. FMD, flow-mediated dilation; cfPWV, carotid-femoral pulse wave velocity; IL-6, interleukin-6; oxLDL, oxidized low-density lipoprotein; BP, blood pressure; Hb A1c' hemoglobin A1c; eGFR, estimated glomerular filtration rate; UACR, urinary albumin-creatinine ratio.
